# Association of x-ray velocimetry (XV) ventilation analysis compared to spirometry

**DOI:** 10.3389/fmedt.2023.1148310

**Published:** 2023-06-22

**Authors:** Jason P. Kirkness, Jonathan Dusting, Nina Eikelis, Piraveen Pirakalathanan, John DeMarco, Stephen L. Shiao, Andreas Fouras

**Affiliations:** ^1^ 4DMedical, Los Angeles, CA, United States; ^2^ Department of Radiation Oncology and Biomedical Sciences, Cedar-Sinai Medical Center, Los Angeles, CA, United States

**Keywords:** ventilation heterogeneity, x-ray velocimetry, regional lung function, ventilation defect percentage, fluoroscopy

## Abstract

**Introduction:**

X-ray Velocimetry (XV) ventilation analysis is a 4-dimensional imaging-based method for quantifying regional ventilation, aiding in the assessment of lung function. We examined the performance characteristics of XV ventilation analysis by examining correlation to spirometry and measurement repeatability.

**Methods:**

XV analysis was assessed in 27 patients receiving thoracic radiotherapy for non-lung cancer malignancies. Measurements were obtained pre-treatment and at 4 and 12-months post-treatment. XV metrics such as ventilation defect percent (VDP) and regional ventilation heterogeneity (VH) were compared to spirometry at each time point, using correlation analysis. Repeatability was assessed between multiple runs of the analysis algorithm, as well as between multiple breaths in the same patient. Change in VH and VDP in a case series over 12 months was used to determine effect size and estimate sample sizes for future studies.

**Results:**

VDP and VH were found to significantly correlate with FEV_1_ and FEV_1_/FVC (range: −0.36 to −0.57; *p *< 0.05). Repeatability tests demonstrated that VDP and VH had less than 2% variability within runs and less than 8% change in metrics between breaths. Three cases were used to illustrate the advantage of XV over spirometry, where XV indicated a change in lung function that was either undetectable or delayed in detection by spirometry. Case A demonstrated an improvement in XV metrics over time despite stable spirometric values. Case B demonstrated a decline in XV metrics as early as 4-months, although spirometric values did not change until 12-months. Case C demonstrated a decline in XV metrics at 12 months post-treatment while spirometric values remained normal throughout the study. Based on the effect sizes in each case, sample sizes ranging from 10 to 38 patients would provide 90% power for future studies aiming to detect similar changes.

**Conclusions:**

The performance and safety of XV analysis make it ideal for both clinical and research applications across most lung indications. Our results support continued research and provide a basis for powering future studies using XV as an endpoint to examine lung health and determine therapeutic efficacy.

## Introduction

Hundreds of millions of people throughout the world suffer from some form of lung disease (1). Diagnosis and monitoring of lung disease relies primarily on pulmonary function tests (PFTs) and imaging, such as x-ray or computed tomography (CT). Chest CT scans can provide qualitative structural information about the lung and are used in the diagnosis of diseases which cause significant structural changes. However, they do not provide any information regarding lung function. PFTs, such as spirometry, do provide functional information for diagnosis and monitoring of lung diseases. It is well known that there may be heterogenous disease expression in patients with COPD, asthma, ILD, CF, and silicosis However, spirometry does not provide any spatial information regarding lung function. Furthermore, both CT and spirometry lack the capability to detect small regional functional changes, which limit existing technology for early-stage disease detection.

X-ray velocimetry (XV) ventilation analysis is a novel technique to directly measure regional ventilation. The analysis quantifies lung tissue motion from fluoroscopy sequences obtained at multiple angles during tidal breathing. More specifically, XV measures the lung tissue's local volumetric expansion at all locations over the entire lung (2, 3). The local volumetric expansion is equivalent to the net increase (or decrease) in volume of air over that time period (4). The local expansion is therefore directly linked to the regional ventilation, which can vary significantly over the lung. Separate measurements of local volumetric expansion are made at all phases within a breath. Using this technique, XV delivers quantitative, local information with high spatial resolution without the need for breath-hold maneuvers or contrast agents. Additionally, since the analysis relies on relatively short fluoroscopy sequences, the radiation dose to the patient is much lower than other lung imaging methods. The radiation dose associated with the fluoroscopic acquisition over the full study is between 2 and 4 standard chest x-rays which equates to 0.2–0.4 mSv. The calculated dose in this study is of the order of 1/1,000th of 1% of the typical adult therapeutic dose of 40 Gy, meaning that the low-radiation dose required to obtain fluoroscopic images for the study added very little risk to the patients.

The relatively low radiation dose and ability to quantify ventilation without contrast or breath-holds makes XV ventilation analysis ideal for many clinical applications. XV ventilation analysis has already been validated through multiple preclinical studies. In rabbits, XV analysis was used to quantify local airflow within the airways, which were highly consistent with values measured using a flowmeter (5, 6). Further studies used XV analysis to investigate lung dysfunction in two mouse models of lung disease. XV analysis showed increased heterogeneity in ventilation throughout the lung in both bleomycin-induced injury and β-ENaC cystic fibrosis models compared to controls (4, 7). Additionally, in both models, local ventilation defects identified via XV corresponded to regions with pathological changes determined via histology.

This study was designed not only to validate the performance of XV, but also to assess its utility for monitoring small changes in regional lung function and compare to gold-standard pulmonary function test (spirometry). We hypothesized that while XV metrics would be generally consistent with gold-standard tests, XV analysis would be more sensitive in providing an earlier detection of functional changes than current tests. Furthermore, this pilot study provides a basis for sample size estimation for future XV-based research.

## Methods

### Study population

**Figure 1:**
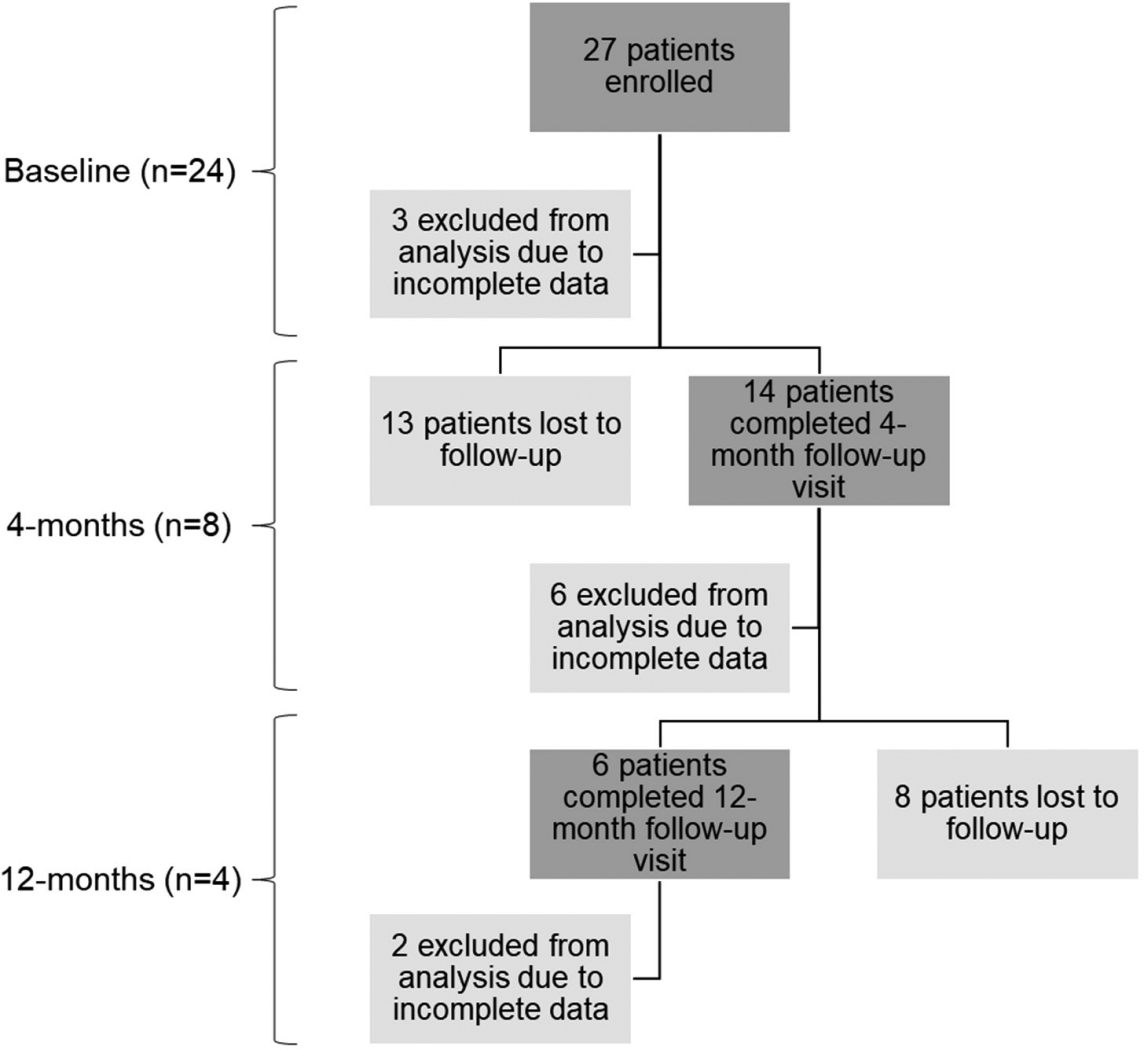
Summary of patient enrollment and sample sizes for each time point. The *n* values represent the final number of patients included in the data analysis for each time point. The baseline time point includes both the screening and pre-treatment visits. Each time point includes a description of the total number of patients, the number excluded from analysis due to incomplete data, and the number lost to follow-up.

This study was approved by the Cedars-Sinai Medical Center Institutional Review Board (NCT02735746). The study included 27 adult patients who were referred to Radiation Oncology at Cedars-Sinai Medical Center for thoracic radiotherapy. Three patients were excluded from the analysis due to incomplete data (see Figure 1).

### Study design

A prospective, longitudinal study was utilized with data collection occurring at three time-points: baseline (*n* = 24), 4-months post-treatment (*n* = 8), and 12-months post-treatment (*n* = 4) (see Figure 1). At each time-point, the collected data included pulmonary function tests (PFTs), thoracic CTs and fluoroscopic lung imaging for XV analysis.

### Radiation treatment

Patients underwent standard-of-care radiation therapy using the Varian TrueBeam STx linear accelerator system (Varian Medical Systems, Palo Alto, CA). Based on a planning CT, a patient-specific treatment dose plan was determined for the thoracic malignancy (excluding lung cancer). Participants were undergoing treatment requiring radiation therapy of a cancerous lesion in the chest wall, breast, or mediastinum. The radiation field included a portion of lung, but patients with lung cancer malignancies were excluded from the study.

### Pulmonary function tests

Pulmonary function testing was conducted as per the American Thoracic Society guidelines (8) to obtain forced expired volume in one second (FEV_1_), forced vital capacity (FVC) and the ratio of FEV_1_ to FVC (FEV_1_/FVC).

### Imaging

Fluoroscopic lung imaging was performed on the Varian TrueBeam system. The field of view was set to include the entire lung. Fluoroscopic lung imaging were captured during tidal breathing with the participant in a supine position of the following angles: 0° AP (Anterior-Posterior axis), ±36° from AP, and ±72° from AP. All fluoroscopic views had the same center of rotation and captured at least one complete, continuous breath. Automatic Exposure Control (AEC) was utilized to ensure the captured images had the highest level of signal-to-noise ratio. The participants were instructed to breathe normally during the scanning procedure. They were asked not to hold their breath or take deeper/shallow breaths.

### XV analysis and metrics

Proprietary flow velocimetry algorithms measure tissue expansion over the course of a breath. Tissue expansion is then used to calculate regional ventilation at each location (voxel) within the lung. The voxels (8 mm^3^) are equally distributed throughout the entire lung field providing specific ventilation measurements for ∼10,000 local regions. Ventilation is expressed in dimensionless units as specific ventilation, which is defined as the change in volume of a lung region since the start of inspiration (ΔV), divided by the volume of the same region at end-expiration (V_0_). The specific ventilation measurements, normalized to the mean, are presented as a coloured contour map illustrating the spectrum of ventilation measurements during breathing. Red depicts regions of relative low ventilation, green depicts regions of relative average ventilation and blue depicts regions of relative high ventilation. This data is also presented as a histogram, which graphically illustrates the frequency distribution of specific ventilation. The specific ventilation values are normalized to the mean of each individual. This approach accounts for individual differences and provides a standardized baseline.

In addition to visualizing the regional specific ventilation, XV analysis provides several metrics of lung health, including ventilation heterogeneity (VH) and ventilation defect percent (VDP) (9). VH is a non-dimensional indicator of the spread of the ventilation frequency distribution. VH is defined as the inter-quartile range of the specific ventilation divided by the mean specific ventilation. The total VH is calculated using all regional specific ventilation data. VH is further stratified in to small and large scale. The small scale VH (VH-SS) value is the degree of heterogeneity within local regions of the lung calculated after first filtering out large scale variations (i.e., scales larger than 64 mm/2.5 in.). Finally, the large scale VH (VH-LS) is the degree of heterogeneity within larger regions of the lung (e.g., lobar and larger), calculated after first filtering out small scale variations (i.e., scales smaller than 64 mm/2.5 in.). VDP is defined as the percentage of lung with a specific ventilation below 60% of the mean specific ventilation.

### Data analysis and statistics

To validate the repeatability of XV outputs, we performed two assessments on a subset of 12 data sets. One data set consists of 5 fluoroscopic sequences acquired at distinct angles during tidal breathing. First, comparison of the results obtained through repeated analysis of the same fluoroscopic lung image sets was undertaken (i.e., run-to-run repeatability). The outputs of the analysis vary slightly between each execution (or ‘run’) of the algorithm. To determine the extent of this variability, the 12 data sets were processed five times each with constant input data and the mean standard deviation was reported. The second repeatability test assessed variability in the results related to natural breath-to-breath variations (i.e., breath-to-breath repeatability) which measures the variance between measurements of the same patient state under the same condition. This test utilized the same 12 data sets in which a patient had at least two breaths captured under identical fluoroscopy settings. With these data sets we determined the mean difference in the metrics between breaths in the same patient.

To compare XV metrics with spirometry outputs, we calculated the Pearson correlation coefficients between XV metrics (VH and VDP) and spirometry outputs (FEV_1_, FVC, and FEV_1_/FVC). Individual cases were used as representative examples of the changes in XV metrics over time. Only those participants who completed all study visits were chosen as the case series. Sample size estimates were based on the change in metrics from baseline to 12-months. Estimates were determined via power analysis for paired *t*-test (*α *= 0.05, power = 0.9). All statistical analyses were performed using XLSTAT V2020.1.3 (Addinsoft, NY).

## Results

**Figure 2:**
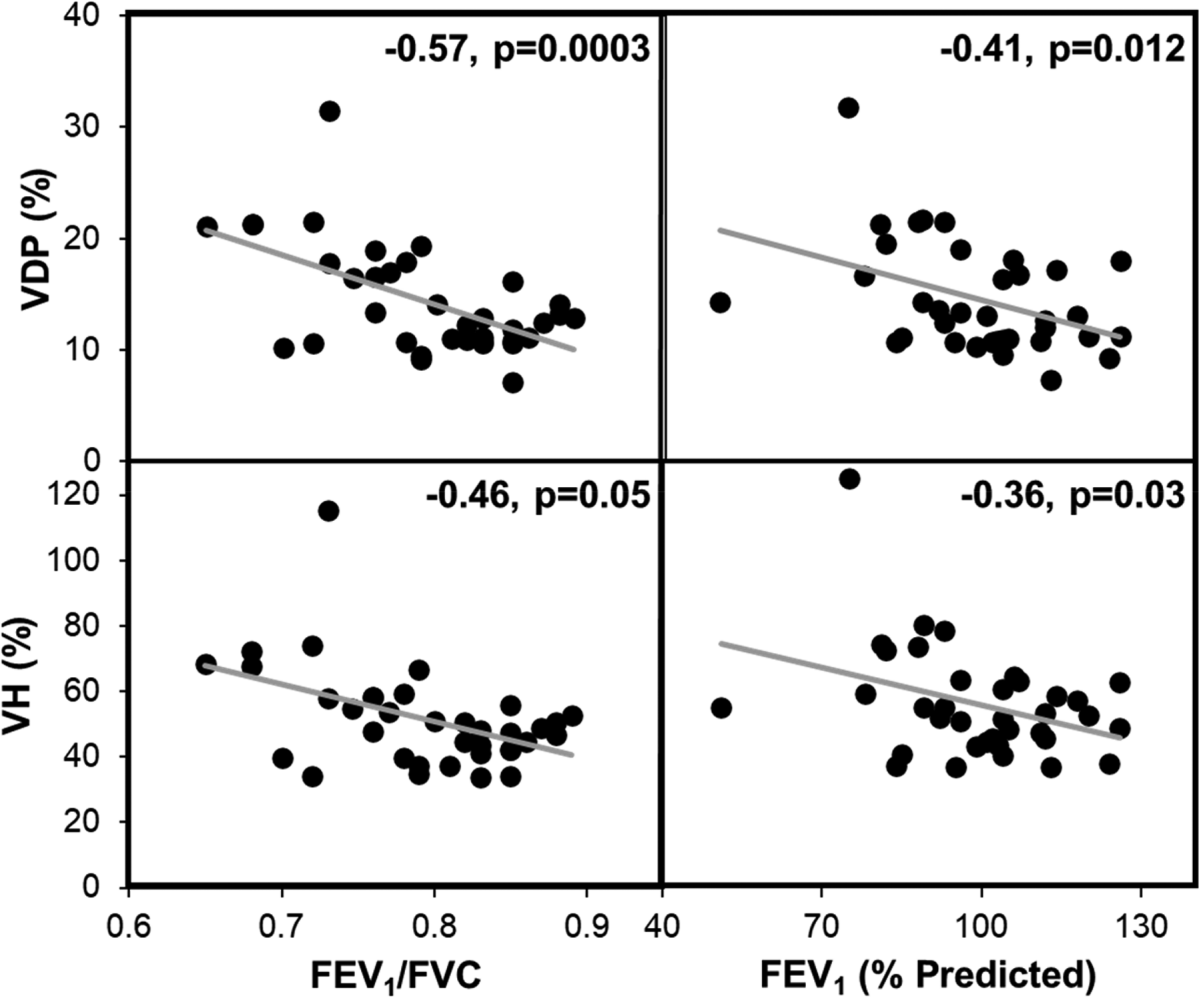
XV metrics VH and VDP significantly correlated with FEV_1_ and FEV_1_/FVC. These plots represent the combined spirometry and XV metrics for each patient at baseline, 4-months, and 12-months (*n* = 36). The gray line represents the linear regression. Pearson correlation coefficients and *p*-values are reported for each plot.

Demographics and baseline characteristics of the study population are summarized in Table 1. Most patients had normal spirometry results at baseline. Correlations between XV metrics (VH and VDP) and spirometry (FEV_1_ and FEV_1_/FVC) were examined and found to show weak to moderate, negative correlations (see Figure 2).

**Table 1:** Summary of patient demographics and baseline characteristic**s.**

Metric	Median (IQR)
Age (years)	59 (51–76)
FEV_1_ (% predicted)	102 (93–112)
FVC (% predicted)	98 (92–112)
FEV_1_/FVC	0.8 (0.76–0.85)
TV (L)	0.32 (0.24–0.41)
Total VH (%)	48.9 (41.1–57.8)
VH-SS (%)	26.8 (24.1–31.3)
VH-LS (%)	28.0 (21.9–37.7)
VDP (%)	12.9 (10.7–16.6)

The results of the repeatability tests are shown in Table 2. The run-to-run variation was less than 2% for all metrics. The mean difference in outputs between breaths ranged from 1.7%–7.8%. Change in XV metrics from baseline to 12-months post-treatment was assessed in the subgroup of patients who completed all study visits. We chose several cases from this group that particularly highlighted the advantages of XV over PFT.

**Table 2:** Variation of XV metrics over multiple XV reconstructions and breaths.

	Run-to-run variation (Mean standard deviation)	Breath-to-breath variation (Mean difference)
Tidal volume	3.7 ml (1.3%)*	20 ml (5.7%)*
Ventilation heterogeneity	1.9%	5.4%
Small-scale ventilation heterogeneity	1.0%	2.5%
Large-scale ventilation heterogeneity	1.7%	7.8%
Ventilation defect percentage	0.6%	1.7%

* Percentage of mean tidal volume measured across all cases.

**Figure 3:**
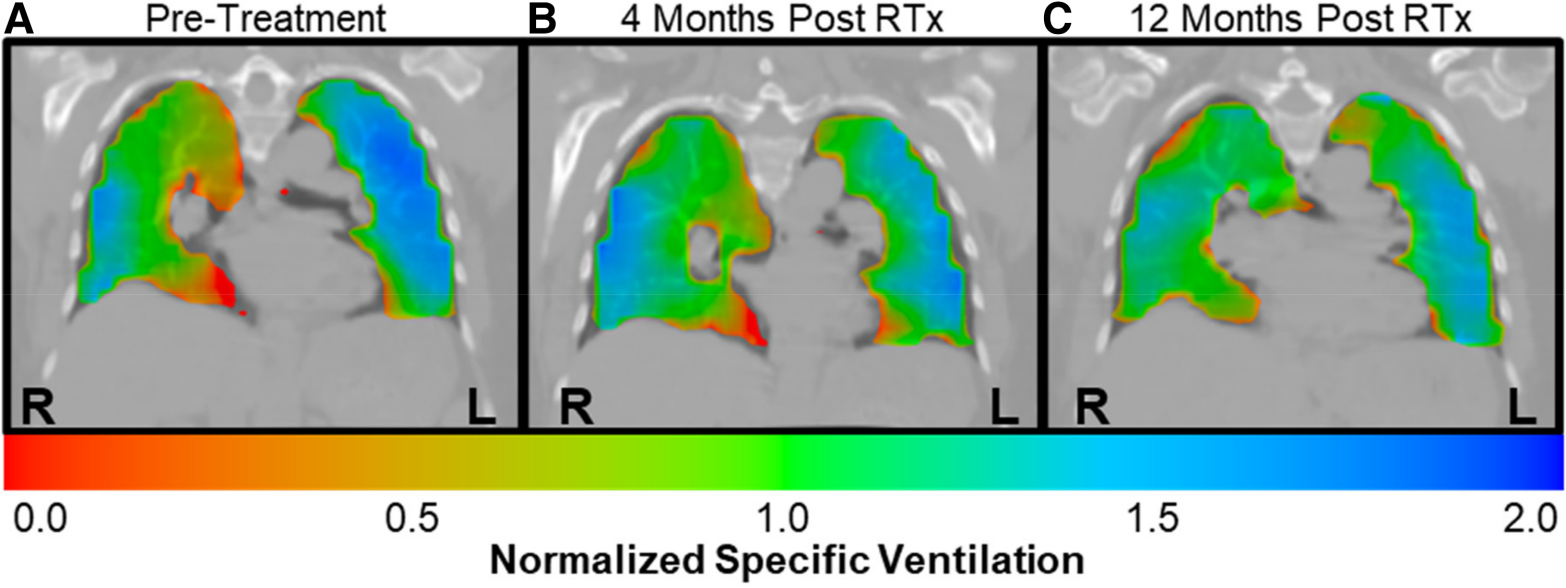
Case A XV ventilation visualization showed decreased ventilation heterogeneity over time. Ventilation visualizations represent the specific ventilation (normalized to the mean) throughout the lungs. Red represents areas with lower than average ventilation, green represent average ventilation, and blue represents higher than average ventilation. (**A**) The pre-treatment ventilation visualization for case A showed considerable heterogeneity, with regions of defect as well as higher than average ventilation throughout the lungs. (**B**) By 4-months post-treatment, the areas of red and blue had decreased, signifying reduced heterogeneity. (**C**) By 12-months post-treatment, ventilation was more homogeneous, with increased areas of green throughout the lungs.

**Figure 4:**
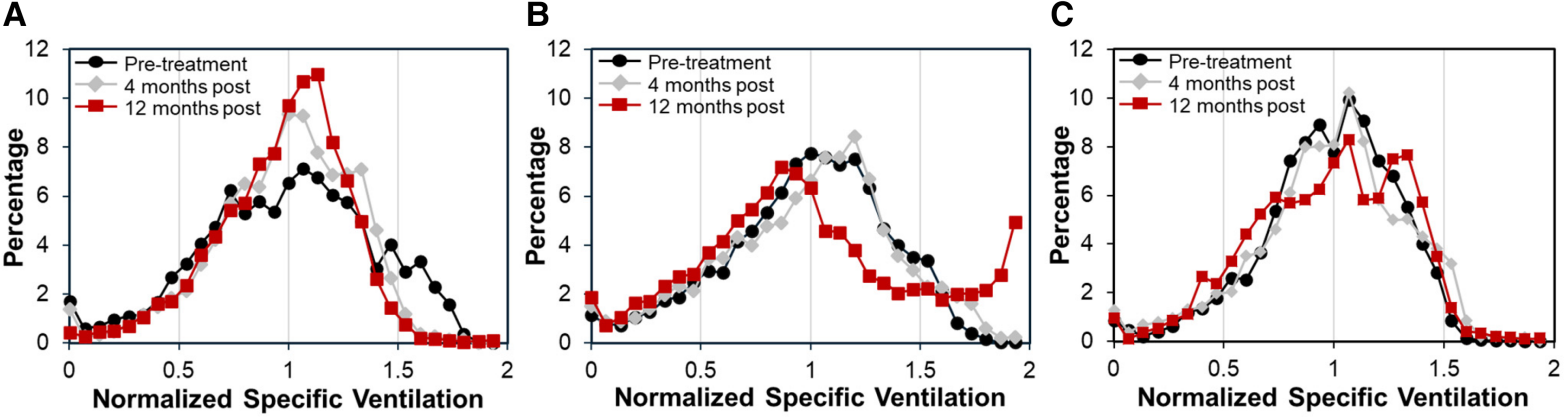
Frequency distributions of normalized specific ventilation illustrated changes in ventilation in each case over time. (**A**) Case A had a relatively wide distribution at pre-treatment (black circles) that narrowed at 4-months (gray diamonds) and further narrowed at 12-months (red squares), demonstrating decreased ventilation heterogeneity over time. (**B**) Case B had a similarly wide distribution at pre-treatment that showed little change at 4-months. However, by 12-months the distribution had shifted significantly, showing an increase in the frequency of over-ventilated regions. (**C**) Case C had a relatively tight distribution pre-treatment that shifted slightly at 4-months. By 12-months the distribution had widened, showing two peaks with one centered at higher than average specific ventilation.

Case A (see Table 3) highlights an improvement in lung function as measured by XV (i.e., VH and VDP decreased by 18.3% and 6.0%, respectively) over the course of the study, whereas PFT remained largely unchanged (i.e., FEV_1_ and FVC remained >100% predicted and FEV_1_/FVC dropped slightly to 0.79 but within the normal range). Figure 3 visually depicts the progressive reduction in spatial regions of defects and heterogeneity at 4- and 12-months post-treatment, which correlates with the quantitative improvement in lung function as seen with the XV metrics. Further, the frequency distribution of specific ventilation values throughout the lung progressively narrowed throughout the study (Figure 4A), demonstrating decreased heterogeneity.

**Table 3:** Longitudinal case series of patients monitored using spirometry and XV analysis, while undergoing thoracic radiation therapy.

Months post-treatment	Case A			Case B			Case C		
	0	4	12	0	4	12	0	4	12
FEV_1_ (% expected)	>100	>100	>100	>100	>100	81	>100	>100	>100
FVC (% expected)	>100	>100	>100	>100	>100	91	>100	>100	>100
FEV_1_/FVC	0.87	0.83	0.79	0.76	0.77	0.65	0.83	0.85	0.89
VH (%)	55.4	43.4	37.1	48.8	53.9	68.3	38.6	42.0	52.7
VH-SS (%)	29.5	26.8	23.3	29.2	29.5	33.6	22.7	26.8	19.1
VH-LS (%)	42.0	22.2	14.9	27.0	33.3	47.1	18.6	31.4	44.7
VDP (%)	15.4	10.7	9.4	15.1	17.0	21.1	10.2	11.9	12.9

Case B (Table 3) demonstrates the sensitivity of XV analysis to detect earlier changes in lung function compared to PFT. Spirometric measurements showed normal lung function at baseline and at 4-months post-treatment (FEV_1_ and FVC were >100% predicted and FEV_1_/FVC was 0.76–0.77), and thereafter a decline in lung function at 12-months post-treatment (FEV_1_ and FVC were 81% and 91% respectively, and FEV_1_/FVC was 0.65). XV analysis detected declining lung function as early as 4-months post-treatment (VH and VDP increased by 5.1% and 1.9% from baseline, respectively), which further progressed by 12-months post-treatment (VH and VDP increased by 19.5% and 6.0% from baseline, respectively).

Case C (Table 3) provides an example where XV analysis detected a moderate, progressive decline in lung function over 12-months (VH and VDP increased by 14.1% and 2.7% from baseline, respectively), while PFT remained consistently normal (FEV_1_, FVC and FEV_1_/FVC were >100% predicted).

Cases A-C serve to demonstrate the capability of XV analysis to detect subtle and/or early changes in lung function, which are otherwise undetectable or delayed using spirometry.

The relative effect sizes observed in the above cases allow for an estimation of sample sizes for future studies. The effect size was observed to range from moderate (VH and VDP changes of 14.1% and 2.7% respectively) to large (VH and VDP changes of −18.3 to +19.5% and ±6.0% respectively). Utilizing a 90% power calculation, sample standard deviations of 16.9% for VH and 5.0% for VDP, and the aforementioned effect sizes, the estimated sample sizes for detecting moderate and large changes in lung function using XV with sufficient power are 17–38 patients and 10–11 patients, respectively.

## Discussion

The validation of performance of XV analysis has now been reported. The methodology for determining ventilation distribution, VDP and VH are repeatable. There was minimal variation between runs of the algorithm on the same dataset. Further, breath-to-breath variability was less than 8% for all metrics. The primary quantitative outputs, VH and VDP, both correlated with FEV_1_ and FEV_1_/FVC. This demonstrates the consistency of XV metrics with current standard-of-care diagnostics. However, the correlations were moderate at best, likely due to the enhanced level of detail provided by XV compared to spirometry. In addition, a strong correlation was not expected since XV metrics are derived from more detailed regional data than is available from spirometry. Indeed, this was illustrated in several cases in which XV analysis detected changes in lung function that were either undetectable or delayed in detection by spirometry.

Regional functional measurements obtained via imaging provide more sensitive spatial information that cannot be obtained from PFT due to temporal and spatial limitation (10). Regional functional data can be obtained through various methods in addition to XV, such as hyperpolarized MRI (11–13), CT densitometry (14–16), or single-photon emission computed tomography (SPECT) (17–19). However, these techniques involve imaging at various stages of the breath while the patient performs breath-hold maneuvers. While this allows inferences to be made about regional airflow, it does not directly quantify air movement. Furthermore, most of these techniques require inhalation of a contrast agent. This not only increases the risk to the patient, but also requires additional time, cost, and effort to produce and contain these potentially harmful contrast agents. Four-dimensional CT (4DCT) is a newer approach that tries to overcome these issues, allowing clinicians to qualitatively observe organ motion (20–22). Image acquisition is gated throughout the breath to aid reconstruction. While 4DCT does not require breath-hold maneuvers, the video processing methods, patient movement, and imperfect gating can introduce artifacts that reduce data quality. As 4DCT requires radiation exposures greater than a standard CT, the radiation dose from this procedure can be a major concern (23). XV addresses the limitations of other methods (24). Here we have validated the performance of XV in providing repeatable, quantitative regional ventilation data through low-dose, free-breathing, contrast-free imaging.

VH and VDP are related to disease severity in a number of conditions. For example, they are increased in patients with COPD (24), cystic fibrosis (25, 26), and asthma (27, 28) compared to healthy controls. Furthermore, higher VH and VDP correlate with lower spirometry metrics (29, 30), increased airway hyperresponsiveness (31), poorer treatment response (32), lower quality of life (24), and more severe outcomes (33). Thus, these metrics are promising biomarkers to support diagnosis, and predict treatment responses and disease outcomes. Additionally, XV analysis provides a promising efficacy endpoint for clinical trials. In this paper we performed sample size estimation to power development of clinical trials. Based on these results, XV provides an opportunity to reduce sample sizes when used as an endpoint compared to spirometry. It is reported that 90% of subjects can reproduce FEV_1_ within 120 ml (6.1%), FVC within 150 ml (5.3%), and Peak Expiratory Flow (PEF) within 0.80 L/min (12%). As such, in a recent COPD trial of tri-therapy (ICS/LABA/LAMA), 3,366 subjects were required in the tri-therapy treatment arm to demonstrate a 7.4% increase in FEV_1_ (34, 35). Large sample sizes increase the cost and extend the duration of such trials. XV analysis is capable of detecting even moderate changes in function, such as in Case C, with relatively small sample sizes. Significant improvements or declines in lung function, such as in Case A and Case B respectively, can be achieved with even smaller samples. Even if there is no observed clinical effect, XV analysis could help determine this with fewer patients in less time and with lower costs.

The performance and safety of XV analysis makes it ideal for both clinical and research applications across most lung indications. Here we assessed XV in patients receiving thorax radiation therapy, but acknowledge that additional validation is required across a range of lung conditions and indications. Our results support continuing research and provide a basis for powering future studies. Furthermore, the sensitivity of XV supports its use as an endpoint in future clinical trials examining changes in lung health and determining therapeutic effect size.

## Data Availability

The raw data supporting the conclusions of this article will be made available by the authors, without undue reservation.
